# Magnitude of Depression and Associated Factors in Women Living With HIV in Northwest, Ethiopia: Mediation Analysis

**DOI:** 10.1155/arat/9578192

**Published:** 2025-01-09

**Authors:** Tadele Amare Zeleke, Tadesse Awoke Ayele, Zewditu Abdissa Denu, Lillian Mwanri, Telake Azale

**Affiliations:** ^1^Department of Psychiatry, College of Medicine and Health Science, University of Gondar, Gondar, Ethiopia; ^2^Department of Epidemiology and Biostatics, Institute of Public Health, College of Medicine and Health Sciences, University of Gondar, Gondar, Ethiopia; ^3^Department of Anesthesia, College of Medicine and Health Sciences, University of Gondar, Gondar, Ethiopia; ^4^Research Center for Public Health, Equity and Human Flourishing, Torrens University Australia, Adelaide Campus, Adelaide 5000, South Australia, Australia; ^5^Department of Health Promotion and Behavioral Sciences, Institute of Public Health, College of Medicine and Health Sciences, University of Gondar, Gondar, Ethiopia

**Keywords:** depression, mediation analysis, social support, WLWHIV

## Abstract

**Background:** Depression in women living with HIV (WLWHIV), is one of the most common public health concerns worldwide. Depression has a negative impact on antiretroviral therapy (ART) adherence, quality of life, poor HIV treatment outcomes, and mortality. However, there is a paucity of evidence in low-income countries such as Ethiopia in WLWHIV.

**Objective:** The aim of this study is to assess the magnitude of depression and related factors, and how social support mediates HIV-related stigma and depression in WLWHIV.

**Method:** A cross-sectional study was conducted among 1043 patients in a health institution, employing a systematic random sampling technique to select the study participants. The structured Patient Health Questionnaire (PHQ-9), Oslo Social Support Scale, Perceived HIV-related stigma scale, Household Food Insecurity Access Scale (HFIAS), and Violence Against Women Scale were used to measure depression, social support, stigma, food insecurity, and intimate partner violence, respectively. Descriptive statistics were computed, and multivariate logistic regression and mediation analyses were conducted to identify factors associated with depression and how they mediate it.

**Results:** The prevalence of depression among WLWHIV was 41.7% (95% CI: 38.7% and 44.5%). Being single (AOR = 1.80, 95% CI: 1.09–2.99), divorced (AOR = 1.56, 95% CI: 1.11–2.19), widowed (AOR = 1.93, 95% CI: 1.31–2.84), experiencing medical illness comorbidity (AOR = 2.74, 95% CI: 1.75–4.30), having a high viral load (AOR = 1.86, 95% CI: 1.00–3.45), receiving social support (AOR = 0.90, 95% CI: 0.84–0.96), experiencing perceived HIV-related stigma (AOR = 1.04, 95% CI: 1.02–1.06), experiencing food insecurity (AOR = 1.07, 95% CI: 1.03–1.11), and experiencing psychological violence (AOR = 2.05, 95% CI: 1.30–3.23) were significantly associated with depression. Social support partially mediated the relationship between perceived HIV-related stigma and depression.

**Conclusion:** More than two of five WLWHIV developed depression. Depression is indirectly affected by perceived HIV-related stigma through social support. Social support enhances mental health well-being.

## 1. Introduction

Depression, particularly in women, is among the most prevalent, chronic, and significant possible causes of disability worldwide [1–3]. The prevalence of depression among women has been reported to be as high as 20% [4], and women are approximately twice as likely as men to experience depression during their lifetime [5]. Although it is not clear why women are disproportionately affected by depression, potential explanations are that women (i) are the primary carers for family members but do not care for themselves, (ii) have lower household income than men, (iii) have less education, (iv) lack social support, and (v) are frequently abused by their counterparts [6]. Women are also more vulnerable to depression than the general population because of changes in the endocrine control of the reproductive system, the changes that are amplified during the menstrual cycle, parturition, and menopausal period [7].

Among mood disorders, depression is the most prevalent psychiatric disorder associated with HIV, with a twofold greater likelihood of risk in PLWHIV than in their counterparts. Depression is more common in people who (i) have not disclosed their HIV status to others, (ii) have lost their family to HIV, (iii) have experienced treatment failure, and (iv) have advanced HIV clinical stages [8]. In developed countries and across the world, the prevalence of this psychiatric disorder among PLWHIV patients is increasing, especially in women, and there are challenges in treating it [9].

When we compare the prevalence of depression among WLWHIV versus men living with HIV was 45% versus 18.6%, respectively, in Mexico [10]. One study reported that the mean depression and anxiety in women are greater than in men [11]. A systematic review reported that the prevalence of depression is more in WLWHIV than men living with HIV and women versus men was 32% versus 22%, respectively [12]. Perceived stigma is more severe in women than in men, which leads to depression [13]. Women are more likely to experience socioeconomic disadvantages such as being less educated, unemployment, and lower income levels [10].

The prevalence of depression among WLWHIV at baseline for cohort study was reported to be 67% in the United States [14]; 58% in the rural United States among WLWHIV [15]; 32% in the United States, after following the study participants; 75% or more in their study visit in the cohort [16]; 15.9% in Florida [17]; and 19.4% in North Central Florida [18]. In South America, the prevalence of depression in WLWHIV has been reported to be 25.8% in Brazil [19], 68% in Peru [20], and 44.7% in Mexico City [10]. In other settings, the prevalence of depression in WLWHIV has been reported to be 44.2% in Indonesia [21] and 25% in the Philippines [22].

The reported prevalence of depression in India has varied, including 51.1% in one study [23] and 34.5% [24] and 46.99% [25] in other studies. In Africa, the prevalence of depression among WLWHIV was reported to be 57.8% in Tanzania [26], 81% in Rwanda [27], 56% [28] and 23.7% [29] in studies in Uganda, and 32.5% in Ethiopia [30].

Depression and HIV-related stigma have been reported to be interrelated [31] and bidirectional, with both depression and stigma being highly correlated among PLWHIV and in turn affecting treatment outcomes [32, 33]. Notably, women are highly vulnerable to both HIV stigma and psychological distress [34]. Although the chronic burden of HIV enhances the risk for depression over time in WLWHIV, this risk can arguably be ameliorated with good social support and reduced stigma [35]. In LMICs, mental health conditions are known to be highly prevalent; however, assessments and interventions are also needed less often [36]. When women with HIV are diagnosed, they develop a sense of sadness, fear, despair, pain, and unpleasant feelings that can lead to chronic depression [37], which is often not related, diagnosed, or treated effectively as a mental health problem [38].

HIV affects the brain, which can be the cause of serotonin reduction, further leading to depression [39]. Depression has multiple impacts on WLWHIV, such as morbidity [17], missing medical appointments, poor antiretroviral therapy (ART) adherence [40], virologic failure [41–43], and poor quality of life. These factors can hasten HIV disease progression, poor organ functionality, and increased mortality [44, 45]. Socially, individuals who are depressed have a greater probability of being unemployed [46]. It has also been estimated that people with depression lose more than 5.6 h of productivity per week [47], costing between $43 billion for medical care services and $17 billion in productivity loss each year [48]. In 2022, the WHO estimated that 12 billion working days were lost every year due to depression and anxiety, costing approximately US$1 trillion per year in lost productivity [49].

Depression can be exacerbated by stigma, intimate partner violence (IPV) [50], lack of disclosure of HIV status, multiple losses [9], and poor social support [26, 51]. People living with HIV who had a high level of social support reported less HIV-related stigma and depression [31]. HIV-related stigma can lead to feelings of isolation, fear, and shame, all of which are closely linked to depression. Women living with HIV (WL WHIV) encounter numerous challenges due to societal discrimination and negative perceptions, which further exacerbate their mental health struggles [52]. Social support serves as a mediator between HIV-related stigma and depression. Women who perceive strong social support are better equipped to cope with the emotional toll of stigma, resulting in lower levels of depression [53]. Social support can empower women to challenge negative stereotypes and beliefs about HIV, leading to a decrease in internalized stigma, which in turn directly lowers the levels of depression [54]. Supportive relationships can cultivate a sense of belonging and acceptance, reducing the feelings of shame and guilt associated with living with HIV [55].

The factors associated with depression in WLWHIV were being unmarried, being widowed [23], and being divorced [11]. In addition, a low educational level [56], rural residence [23, 24], and having five or more children were factors associated with depression [57]. Having a history of abortion, preterm birth [58] and stillbirth [59] were additional risk factors for depression. Depression can also be affected by WHO clinical stages of HIV II and III [29, 30], increased viral loads [29, 30, 51], coinfections [27, 30], poor drug adherence [51] and a duration of illness of 4 years or more [60]. Drug abuse, including alcohol use [29, 61], khat chewing, and smoking [29], is a potential predictor of depression [62].

However, to the best of our knowledge, no prior studies in Ethiopia have explored whether social support is a mediator of the relationship between perceived HIV-related stigma and depression or factors affecting depression among WLWHIV. Therefore, the aim of this study was to assess the magnitude and associated factors of depression among WLWHIV in Gondar town health facilities, in northwest, Ethiopia.

## 2. Methods and Materials

### 2.1. Study Area and Period

The current study was conducted in health facilities in Gondar town. Gondar is located in northwestern Ethiopia, approximately 728 km away from the capital of the country, Addis Ababa. In Gondar town, there is one comprehensive specialized hospital, one primary hospital, and eight health centers, namely, the University of Gondar Comprehensive Specialized Referral Hospital ((UoGCSRH), Ayra Primary Hospital, and Azezo, Maraki, Poly, Mintwab, Gebrael, Teda, Bilajig, and Woleka Health Centers. Except for BIlajig Hospital, all health facilities provide ART services. There were approximately 6042 adult WLWHIV who had registered for ART follow-up in Gondar town health facilities during the study period from September 1st to October 30^th^, 2023, with the majority attending the UoGCSRH, the largest facility among all, and the only hospital that gives mental health services at outpatient department (OPD) level and inpatient with the profession of a psychiatrist, psychiatry nurses, and clinical psychologist, and providing ARTS in four OPDs. The total number of PLWHIV attending the UoGCSRH was 5543, of which 3504 were females and 3355 were WLWHIV aged 18 years or older.

For this study, four health facilities were selected based on the patient flow. These are the UoGCSRH, Azezo Health Center, Gondar/Poly Health Center, and Maraki Health Center.

### 2.2. Study Design

A health institutional cross-sectional study design was used to assess the magnitude and associated factors of depression among WLWHIV attending an ART clinic.

#### 2.2.1. Study Population

The study population included all adults aged ≥ 18 years, women who were HIV-positive, and those who received ART treatment at health facilities in Gondar town. The WLWHIV ages 18 and above were 559 in Azezo, 357 in Maraki, 1521 in Gondar/Poly, 178 in Teda, 42 in Gebrael, 21 in Woleka, and 9 in Mintwab.

### 2.3. Inclusion and Exclusion Criteria

#### 2.3.1. Inclusion Criteria

The study comprised women who were receiving ART for 6 months and above.

#### 2.3.2. Exclusion Criteria

WLWHIV who were unable to communicate and had a serious medical illness were excluded.

#### 2.3.3. Sample Size Determination and Sampling Technique

A single population proportion formula was used to calculate the sample size, by considering 95% confidence, 3% marginal error, and 32.5% prevalence of depression among WLWHIV [30]. Ten of the nonresponses were added, and then the total sample size was 1050 women.

**Figure 1:**
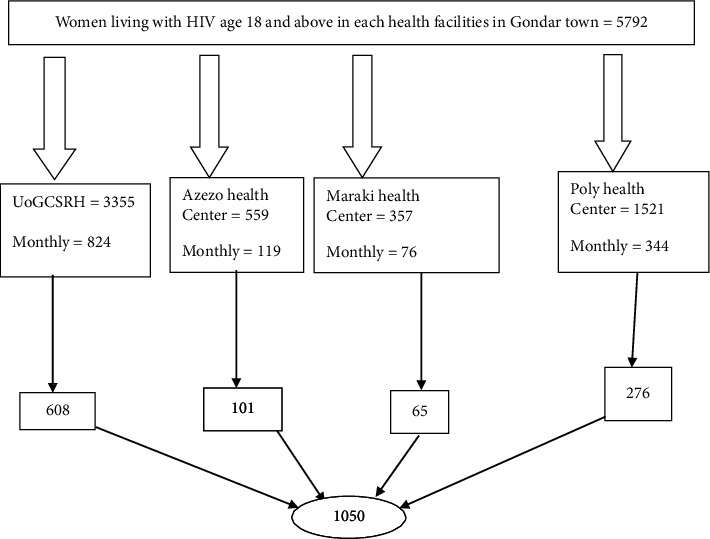
Schematic presentation of the sampling technique and proportion allocated among WLWHIV in Gondar town, Northwest Ethiopia, 2023.

To participate in the study, potential participants were selected using systematic random sampling. The sampling interval (*k* = 2) was calculated by dividing the number of women on ART (1363) each month during data collection by the total sample size (1363/1050 = 1.3 ≈ 2). The total sample size was 1050, and the first woman was selected by the lottery method from the first two ART women; at that time, every two women were included in the study. For each health facility, the sample size was proportionally allocated (Figure 1).

### 2.4. Variables of the Study

#### 2.4.1. Dependent Variables

The dependent variable included the following: depression (yes/no).

#### 2.4.2. Independent Variables

Sociodemographic factors included age, educational status, marital status, living arrangement, residency, distance, and monthly income.

Clinical factors included WHO clinical stage of AIDS, viral load, opportunistic infection, comorbid medical illness, family history of mental illness, drug adherence, drug side effects, and duration of illness.

Psychosocial factors include social support, IPV, HIV status disclosure, and stigma. Behavioral factors were ART adherence, alcohol, cigarettes, and chat.

### 2.5. Operational Definition

Depression was defined as a score of 10 or above on the PHQ-9 questionnaires [63].

Social support: Women who score according to the Oslo Social Support Scale (poor = 3–8, moderate = 9–11, and strong = 12–14), and as the scale increases, social support increases [64].

Current substance use is defined as the use of alcohol, chats, or cigarettes by a WLWHIV for the last 3 months according to ASSIST [65].

ART nonadherence was defined as a woman who answered at least one yes for Questions 1, 2, 3, or 5 or once or more times missed the medication or 2 or more days missed the medication; this was considered ART nonadherence according to the Simplified Medication Adherence Questionnaire (SMAQ) [66].

Perceived stigma was assessed by 12 items of HIV-related stigma; as the score increased, the stigma increased [67].

IPV was defined as a woman who scored at least one of the 13 items on the World Health Organization's Violence Against Women Scale. There was at least one instance of physical violence in the six items. Psychological violence was indicated by at least one of the four items, and sexual violence was indicated by at least one item [68].

According to the Household Food Insecurity Access Scale (HFIAS), food insecurity increases as the score increases [69].

Patients with known chronic medical illnesses were defined as those who had a known medical illness such as diabetes, hypertension, or epilepsy.

Side effects of ART: Respondents reported side effects, including appetite changes, nausea, vomiting, difficulty sleeping, rash, body shape changes, hair loss, numbness in the limbs, fatigue, pain in the limbs, and vision changes, in their medical reviews.

### 2.6. Data Collection Tool and Procedure

Semistructured interviewer–administered questionnaires were used for data collection. Depression was measured by the nine-item Patient Health Questionnaire (PHQ-9), with each item requiring participants to rate the frequency of depressive symptoms experienced 2 weeks before the data collection period. The total score ranged from 0 to 27. The severity of depression was assessed using a four-point Likert scale ranging from 0 to 3, with depression levels measured as follows: no depression (*0–4 points*), mild depression (*5–9 points*), moderate depression (*10–14 points*), moderately severe depression (*15–19 points*), and severe depression (*20–27 points*) [70]. In a validity study of the PHQ-9 in primary healthcare followers, the sensitivity and specificity were 77.8 and 80.6, respectively, and the negative predictive value was 98.3 at a cutoff score of 6 and above [71]. In a study that validated the PHQ-9 among primary health followers aged 18 years and above, with cutoff points of 10 and above, the sensitivity and specificity were 86.2% and 67.3%, respectively, with an overall Cronbach's alpha of 0.81 [63]. In Ethiopia, Jimma University Specialized Hospital, among people living with HIV, the PHQ-9 was validated at a cutoff point of 5/6, with a sensitivity of 87.2%, a specificity of 83.7%, and a Cronbach's alpha of 0.95 [72]. In Kenya, the PHQ-9 was validated among PLWHIV, and the overall reliability was 0.78 [73].

The SMAQ is a self-administered questionnaire that consists of six items, of which four are “yes/no (*dichotomous*)”, one is a Likert-type questionnaire, and one has two options: (1) “Do you ever forget to take your medicine? (i) Yes and (ii) No,” (2) “Are you careless at times about taking your medicine? (i) Yes and (ii) No,” (3) “Sometimes if you feel worse, do you stop taking your medicines? (i) Yes and (ii) No,” (4) “Thinking about the last week, how often have you not taken your medicine? (i) Never, (ii) 1–2 times, (iii) 3–5 times, and (iv). > 5 times,” (5) “Did you not take any of your medicine over the past weekend? (i) Yes and (ii) No,” and (6) “Over the past 3 months, how many days have you not taken any medicine at all? (i) ≤ 2 days and (ii) > 2 days.” If the participants answered at least one “yes” for Questions 1, 2, 3, and 5 and for Question 4, it means that they once or more times missed the medication, and for Question 6, more than 2 days not taking their medication was considered ART nonadherence [66].

Oslo social support at a time when faced with difficulties and critical conditions such as financial, social, and psychological conditions was assessed by the Oslo-3 Scale tool, which has a total of 14 scores and is classified into three broad categories: poor support (*3–8*), moderate support (*9–11*), and strong support (*12–14*) [64].

The risk of substance use was measured using the modified WHO Alcohol, Smoking, and Substance Involvement Screening Test (ASSIST) Version 3.1, which consists of seven items for alcohol, chat, and tobacco product use. However, for this study, only current substance use items were used. The current substance use assessment inquiries about the participants' consumption of alcohol, chats, or/and tobacco in the last 3 months, with questions asked requiring yes or no responses. The four questions asked were “Have you used any kind of alcohol drinks in the last 3 months?,” “Have you used chat in the last 3 months?,” and “Have you used any kind of tobacco products in the last 3 months?” If the participants responded “yes” to any of the questions, then they had used the substance during the last 3 months [65].

Perceived HIV-related stigma was measured by 12 items, each comprising a four-point Likert scale (strongly disagree, disagree, agree, and strongly agree), with a higher score corresponding to a higher level of stigma. The total score of the scale for PS ranged from 12–48 [67]. IPV was assessed by using the World Health Organization's Violence Against Women Scale. The assessment tool has 13 items with yes or no answers. It has three components: physical violence (six items), psychological violence (four items), and sexual violence (three items). At least one yes for each component would indicate physical, psychological, or sexual violence [68].

Food insecurity was assessed by using the HFIAS, a user-friendly tool for assessing food insecurity. Its reliability and validity have been checked in different countries, including low- and middle-income countries. It has nine items with scores ranging from 0 to 27. The responses for the questions were never = 0, rarely = 1, sometimes = 2, and often = 3 [69].

The questionnaire was originally prepared in the English language and subsequently translated into the local language Amharic by language experts. The Amharic version was translated back to English to verify its consistency. The data were collected using the Amharic interviewer–administered version of the questionnaire by eight trained Bachelor of Science (BSc) psychiatrists. The interviews were held in private rooms near the ART units and patients' chart reviews were held. The collection process was supervised by the three MSc psychiatry professionals and the principal investigator.

### 2.7. Data Quality Control

The questionnaire was pretested for reliability on 5% of the sample size at the Kola Diba Health Center. The reliability of the PHQ-9 in the pretest was measured with a Cronbach's alpha of 0.903. Prior to their participation in data collection, the data collectors were provided 2 days of intensive training informing them about the purpose and objectives of the study, the interviewing technique, and how to complete the questionnaire and data management. The interviewers were also informed about ethical principles, including confidentiality of information and the rights of the participants to withdraw from the study if desired. Every day, the questionnaires were reviewed and checked for completeness, consistency, and relevance by investigators and supervisors.

### 2.8. Data Processing and Analysis

The data were checked, coded, and entered into EpiData Version 3.1 and exported to STATA Version 14 for cleaning, coding, and analysis. Descriptive statistics, including frequencies, percentages, and means/medians with standard deviations or interquartile ranges, were reported in the form of texts and tables. Bivariate regression was performed to determine the associations between the dependent and independent variables. Variables with a *p* value < 0.2 in the bivariate analysis were included in the multivariate logistic regression model. Multicollinearity was checked by using variance inflation factor (VIF) and model goodness of fit was tested by the Hosmer–Lemeshow test. A *p* value < 0.05 was considered to indicate statistical significance, and an adjusted odds ratio (AOR) with a 95% confidence interval (CI) was used. The logistic regression results showed that the selected model was a good logistic regression model fit. The Hosmer–Lemeshow goodness of fit was 0.88, and the *p* value was greater than 0.05. In addition, the pseudo-*R*-square was used if the regression result showed that the square of the correlation between the model's predicted values and the actual values of the outcome of this correlation was 41.8%. The model explained 41.8% (Nagelkerke *R*^2^) of the variance in depression symptoms. In mental health research, a Nagelkerke *R*^2^ value ranges from 0 to 1, and close to one shows the perfect fit of the model. In mental health research, 30% or higher is considered acceptable, as it reflects the challenges inherent in capturing the complexity of human behavior [74, 75].

In the mediation analysis, both perceived HIV-related stigma and social support were continuous variables, and depression was a binary outcome. The hypothesized mediation model was tested using Model 4 in SPSS Version 23, comprising an independent variable (perceived HIV-related stigma), a dependent variable (depression), and a mediating variable (social support). Finally, the study calculated a 95% CI based on bootstrap estimation with 5000 bootstrap samples.

### 2.9. Ethical Considerations

The authors assured that all procedures contributing to this work comply with the ethical standards of the relevant national and institutional committees on human experimentation and with the Helsinki Declaration of 1975, as revised in 2008. This research was conducted in accordance with the guidelines of the Declaration of the University of Gondar and was approved by the Institutional Review Board of the University of Gondar College of Medicine and Health Science with approval number 06/02699/8/2023. Moreover, we obtained written informed consent with a sign or thumbprint from all study participants before starting the investigation. Those who score 10 and above according to PHQ-9 were linked to a psychiatric clinic for further screening and treatment.

## 3. Results

### 3.1. Sociodemographic Factors

The response rate of this study was 99.3% (1043/1050). The mean age (±SD) of the respondents was 41.41 (±10.49), ranging from 18 to 78 years. Of the study subjects, 385 (36.9%) were divorced and 330 (31.6%) were married. The majority of respondents, 929 (89.1%) were orthodox religious followers, 689 (66.1%) had low incomes, 394 (37.8%) had no formal education, and only 107 (10.3%) had a diploma or above (Table 1).

### 3.2. Obstetric-Related Factors

The obstetric characteristics of the respondents are shown in Table 2. One in twenty (51 [4.9%]) had a history of unplanned pregnancy, and 74 (7.1%) had a history of abortion.

### 3.3. Clinical-Related Factors

Of the total respondents, 108 (10.4%) reported comorbid medical illness. Sixteen (1.5%) of the participants had mental illness, and 30 (2.88%) participants reported a family history of mental illness. The types of mental illness included psychosis in 10 patients (1%), generalized anxiety disorder in 5 patients (0.5%), and schizophrenia in 1 patient (0.1%). The median viral load and interquartile range (IQR) were 6 and 11, respectively. The WHO clinical percentages of patients with Stages I, II, III, and IV HIV were 955 (91.65%), 45 (4.3%), 20 (1.9%), and 23 (2.2%), respectively.

For the ART regimen, the majority of the study participants (94, 95.3%) were on first-line ART regimens, 44 (4.2%) were on second-line ART regimens, and 5 (0.5%) were on third-line ART regimens. Only 17 (1.63%) reported ART side effects. There were 317 (30.4%) study participants who reported fear of contracting COVID-19. Seven hundred and fifty (71.9%) participants reported that they disclosed their HIV status to others (Table 3).

### 3.4. Behavioral Factors of the Respondents

Nearly one out of five of the participants (189, 19%) had ART drug nonadherence. For the last three months, 346 (33.2%) participants consumed alcohol, 16 (1.5%) chewed khat, and 2 (0.2%) smoked cigarettes.

### 3.5. Psychosocial Characteristics of the Respondents

The mean scores for social support and perceived HIV-related stigma were 9.2 (SD ± 2.24) and 24.4 (SD ± 6.53), respectively. Social support was categorized into three groups as follows: 580 (55.6%) participants had moderate social support, 331 (31.7%) had poor social support, and 132 (12.7%) had strong social support.

### 3.6. Food Insecurity of the Study Participants

The mean and standard deviation for food insecurity of the study participants were 3.3 (SD ± 4.2).

#### 3.6.1. IPV

**Figure 2:**
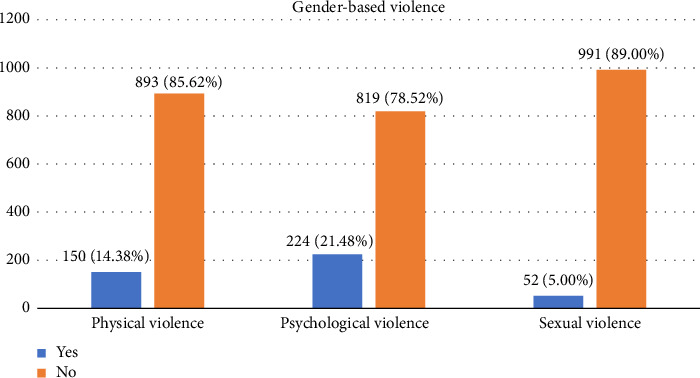
Gender-based violence among WLWHIV in Gondar town, Northwest Ethiopia, 2023.

As demonstrated in Figure 2 above, 224 (21.48%) WLWHIV were psychologically violated, and 52 (5.00%) were sexually violated.

#### 3.6.2. Magnitude of Depression Among WLWHIV

**Figure 3:**
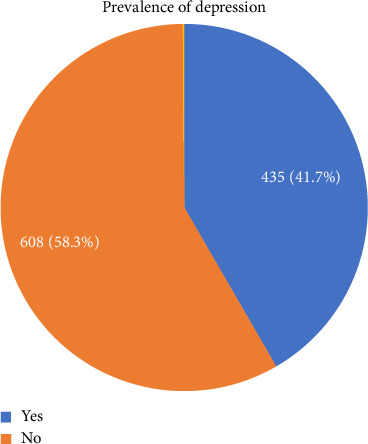
Prevalence of depression among WLWHIV in Gondar town, Northwest Ethiopia, 2023.

In Figure 3 the prevalence of depression was 41.7% (95% CI: 38.7% and 44.5%). The severity of depression was reported as follows: 50.8% (530), 7.5% (78), 31.1% (324), 9.0% (94), and 1.6% (17) for no depression, mild depression, moderate depression, moderately severe depression, and severe depression, respectively.

#### 3.6.3. Factors Associated With Depression in the Bivariate and Multivariate Analyses

According to the bivariate logistic regression, the variables with p values less than 0.2 were marital status, unplanned pregnancy, history of infertility, history of abortion, medical illness, family history of mental illness, viral load, fear of COVID-19, disclosure of HIV status, duration of medication, alcohol use for the last 3 months, social support, HIV-related stigma, food insecurity, physical violence, psychological violence, and sexual violence.

However, in the multivariate logistic regression, the variables with p values less than 0.05 were marital status, medical illness status, viral load, social support status, HIV-related stigma, food insecurity, and psychological violence. Being single had approximately 2 (AOR = 1.80, 95% CI: 1.09–2.99) times greater odds of depression than being married. The odds of depression were approximately 2 (AOR = 1.56, 95% CI: 1.11–2.19) times greater for divorced individuals than for married individuals and approximately two (AOR = 1.93, 95% CI: 1.31–2.84) times greater for widowed individuals than for married individuals (Table 4). The odds of depression were approximately three times greater for respondents who had comorbidities (AOR = 2.74, 95% CI: 1.75–4.30) than for those who did not have comorbidities. The odds of depression were approximately two (AOR = 1.86, 95% CI: 1.00–3.45) times greater among those who had a viral load of 75 copies/mL or more than among those who had a viral load less than 75 copies/mL. When social support increased by one unit, the odds of depression decreased by 10% (AOR = 0.90, 95% CI: 0.84–0.96). A one-point increase in the total perceived HIV-related stigma score was associated with a 4% increase in the odds of current depression (AOR = 1.04, 95% CI: 1.02–1.06). When food insecurity increased by one unit, the odds of depression also increased by 7% (AOR = 1.07, 95% CI: 1.03–1.11). WLWHIV with psychological violence were two AOR = 2.05, 95% CI: 1.30–3.23) times more likely to have depression than those who had no psychological violence (Table 4).

#### 3.6.4. Mediation (Social Support as a Mediator)

The study aims to test if social support mediates the relationship between HIV-related stigma and depression. Initial Pearson r correlation results show that all variables have a strong relationship with each other. Multiple regression was used to test the mediation model.  Table 5 reveals that HIV-related stigma positively predicts depression (*β* = 0.17, 95% CI: 0.097 and 0.248, *p* < 0.001). Furthermore, social support significantly mediates this relationship (*β* = 0.04, 95% CI: 0.024 and 0.061, *p* < 0.001). It is estimated that social support accounts for 23.5% of HIV-related stigma's effect on depression. Nevertheless, HIV-related stigma still has a significant positive direct effect (*β* = −0.13, 95% CI: 0.074 and 0.188, *p* < 0.001).

## 4. Discussion

### 4.1. The Prevalence of Depression

We found that the prevalence of depression among WLWHIV in our study population was 41.7% (95% CI: 38.7% and 44.5%), which is consistent with other findings that depression is the most prevalent psychiatric disorder associated with HIV/AIDS disease, with a twofold greater likelihood of risk in HIV-affected populations than in those who are not infected with HIV [8]. In this study, being single, being divorced, being widowed, having comorbidities, having a high viral load, having low social support, having high perceived HIV-related stigma, experiencing food insecurity, and experiencing psychological violence were significant risk factors for WLWHIV.

The current findings were supported by studies conducted in other countries, such as Indonesia (44.2%) [21] and Mexico City (44.7%) [10]. Informed by the literature and considering our findings, we hypothesize that depression could be the most common psychiatric disorder in HIV-infected individuals in Ethiopia, supporting the global picture [1–3].

Even within Ethiopia, the current findings indicate that the magnitude of depression was greater than that reported in previous studies conducted in this nation, for example, a study at Jigjiga, which reported a prevalence of 32.5% (*n* = 357) [30]. We allude that the current findings were reasonably correct, as the study included four health facilities and used a larger sample size than the previous one. Another study in Kenya showed depression to be less prevalent at 31.6%. This discrepancy could be due to the small sample size [76], which was, however, reported to have been conducted among mothers whose children were vaccinated for HIV, making them happier, with this happiness being a potential mediating factor to reduce depression in these mothers [77]. The tool used for the Kenyan study was the BDI-11, which could have been less reliable, with a Cronbach's alpha of 0.81 [61], in comparison to the current study where the Cronbach's alpha was 0.95 [72]. It is reasonable to argue that sample size and the tools used to assess depression could be among the factors that lead to differences in recorded prevalence. For example, another study in Kenya revealed that the prevalence of depression was 35.5%, with a sample size of 313, whereas studies in other settings, such as in India (*n* = 145), where the prevalence of depression among WLWHIV was 34.5% [24], and in the Philippines (*n* = 150), where the prevalence of depression was 25% [22], suggest the attribution of small samples to the variation in prevalence levels.

Interestingly, in Uganda, a study conducted among WLWHIV reported a lower prevalence (23.7%). The lower prevalence was attributed to women having a high power in sexual relationships [29]. In the US, mental health services are better than in the current study setting, which could also be among the factors that minimized depressive symptoms in WLWHIV, with the prevalence reported in several studies to be 32% [16], 15.9% [17], and 19.4% [18], respectively. However, in addition to the availability of better services in developed settings, the reported lower prevalence could also be attributable to the small sample size (63 and 93) and the type of tools used to measure depression. For example, the DSM-IV has been used to evaluate depression by clinicians in developed settings, which could help to minimize the overlap between somatic symptoms and physical symptoms [17], leading to a better diagnosis of depression than when using the assessment tool [17, 18]. When the DSM-IV was used to evaluate depression by clinicians in Brazil, the prevalence of depression among WLWHIV was also lower at 25.8% [19], which is indicative of a better diagnosis because the use of the DSM- IV could have minimized the overlap of somatic symptoms and physical symptoms that overestimate depression [19].

The sensitivity and specificity of the tools used are also predictive factors for the prevalence of depression among WLWWH. For example, in Tanzania, where they used the HSCL, a tool with a sensitivity and specificity of 88% and 89%, respectively, the prevalence of depression was 57.8% [26]. In another study in the rural United States, the prevalence of depression was 58% [15] despite the above assertion that depression is lower in developed settings due to better health services, indicating that the use of a high-sensitivity assessment tool (CES-D) could be highly prevalent [15].

In the current study, the PHQ-9 has a sensitivity and specificity of 87.2% and 83.7%, respectively [72], with the potential to indicate that the greater the sensitivity and specificity are, the greater the prevalence of depression.

In addition to the sample size and the type of tools used, however, depression can be affected by other contextual and sociodemographic factors and the length of time since the HIV diagnosis was made. For example, in Rwanda, the prevalence of depression was 81% [27] among WLWHIV. We hypothesize that this very high prevalence could have been attributable to factors such as extreme trauma, as the study was conducted among women who had experienced war-related atrocities, and rape had been used as a weapon of war and genocide in some of these women. Women who have been raped have a high likelihood of contracting HIV, experiencing increased genocide-related trauma and posttraumatic syndrome disorder (PTSD), which manifests as depressive symptoms [27]. In Peru, the prevalence of depression was as high as 68%. The reason for this discrepancy might be that in Peru, a study was conducted on impoverished women in which poverty and socioeconomic vulnerability contributed to depression [20]. In Uganda, the prevalence of depression among women aged between 15 and 24 years was 56%, which was higher than the prevalence reported in the present study (41.7%) and a previously conducted study in Uganda in older women [29], confirming the available knowledge indicating that the incidence of major depressive disorders is greater in people younger than approximately 20 years of age [78]. The lengthy period of HIV diagnosis seemed to correspond with increased levels of depression. For example, after the diagnosis of HIV, 65.3% of women in Kenya were exposed to intense and ongoing trauma and were found to have severe depression [79]. A study in India showed a greater prevalence of depression (51.1%) among women who were receiving treatment at a tertiary-level health facility [23], an indication of greater longevity since HIV diagnosis.

### 4.2. Factors Related to Depression

In the current study, the factors related to depression were marital status, medical comorbidities, viral load, social support, perceived HIV-related stigma, food insecurity and psychological violence.

Being single had approximately 2 (AOR = 1.80, 95% CI 1.09–2.99) times greater odds of having depression than being married, which is consistent with the findings of other studies in which single women had a greater level of depression than married women [23, 80]. Women who were divorced had approximately 2 (AOR = 1.56 with 95% CI: 1.11–2.19) times greater odds of depression than married women, an assertion that is supported by other findings [11]. Being widowed had approximately two (AOR = 1.93 with 95% CI: 1.31–2.84) times greater odds of depression than being married, supporting the notion that losing a loved one and/or multiple losses could increase depression levels [8, 9, 23].

In the present study, respondents who had comorbidities had approximately three times greater odds of having depression (AOR = 2.74 with 95% CI: 1.75–4.30) than did those who did not have a comorbid medical illness, a finding that supports other studies in which coinfections have been reported to increase depressive symptoms [27, 30].

The odds of depression were approximately two (AOR = 1.86, with 95% CI: 1.00–3.45) times greater among those who had a viral load of 75 copies/mL or more than among those who had a viral load less than 75 copies/mL. These findings concur with other studies where virologic factors were associated with depression [81] such that the level of viral load corresponded with levels of depression [82]. Higher viral loads have been shown to affect brain function, which correspondingly affects behavioral changes in individuals with depression [29, 30, 51].

Social support was also another factor that was significantly associated with depression. When social support increased by one unit, the odds of depression decreased by 10% [AOR = 0.90 with 95% CI (0.84–0.96)]. This finding was supported by other results in which the quality of social support in HIV-infected individuals, including the size of social networks, was correlated with the level of depressive symptoms [83]. A large body of research has demonstrated that social support minimizes the stress caused by HIV disease [84], resulting in better adaptation outcomes and lower depression. Previous research has also indicated that individuals who have social support feel comfortable, that sharing information among individuals or groups is reported to reduce long-term psychological impairment [85], and that social support is a protective factor against depression in HIV-infected individuals [26, 51, 86, 87].

In this study, perceived HIV-related stigma was significantly associated with depression. An increase in perceived HIV-related stigma per unit increased the odds of depression by 4% (AOR = 1.04 with 95% CI: 1.02–1.06), which is in line with other studies in which increased HIV-related stigma was significantly associated with depression among WLWHIV. To improve depression, treating and reducing HIV-related stigma is needed [88], as stigmatized social identities are subjected to chronic levels of stress and affect the disclosure of HIV status to others [89]. Stigma reduces access to both medical and mental healthcare services. HIV- related stigma causes isolation and loneliness [90] and has a negative impact on psychological well-being [87]. People who experience stigma are more likely to miss clinic visits and experience difficulty taking ART, further exacerbating depression [82]. After being diagnosed with HIV, women experience stigma, which leads to social isolation, loneliness, and depression [6].

A one-unit increase in food insecurity increased the odds of depression by 7% (AOR = 1.07 with 95% CI: 1.03–1.11), findings supported by other studies that describe persistent food insecurity in placing WLWHIV at risk of depression and poor mental health well-being [91]. Other studies have reported that food insecurity significantly exacerbates depression in WLWHIV [92] and that improvements in food security improve mental health [93].

WLWHIV with psychological violence were two (AOR = 2.05, 95% CI: 1.30–3.23) times more likely to have depression than those who had no psychological violence, and emotional abuse was associated with depression [94]. Psychological violence was the most common, followed by physical and sexual violence, which affected marital status and mental illness [95]. Psychological violence is common among married women and is independently associated with depressive episodes [76]. IPV negatively affects women's physical, mental, and social well-being [96], and it exacerbates interactions between HIV and mental health problems, poverty, and stigma [97]. In African women, both IPV and HIV are strongly associated with increased common mental disorders [97]. Women's abuse by adult partners, psychologically violates them, increases their vulnerability to social discrimination, poorer health status, and reduced access to critical resources [98], resulting in complex outcomes such as exacerbating and/or precipitating mental health crises, leading women to miss clinic visits and have difficulties taking ART, further causing depression [82]. The relationship between violence and depression persists even after controlling for all other factors [99] because psychological violence is a traumatic experience that is strongly associated with depression [50].

In combination, perceived HIV-related stigma and social support have synergetic effects on predicting depression [100]. The positive relationship between perceived HIV-related stigma and depression may be mediated by the level of social support [101]. Perceived HIV-related stigma can prevent WLWHIV from seeking and receiving social support, which can lead to depression. Depression, in turn, affects relationships with others and deters their treatment-seeking and deterioration of health [87]. Perceived social support was associated with fewer depressive symptoms, and less stigma and social support mediated the impact of stigma on depressive symptoms. This suggests that social support intervention is important for reducing the impact of stigma on poor psychosocial health outcomes [54]. Increased social support decreases the negative impact of HIV-related stigma [52]. The stigma of HIV-infected individuals may reduce the quality of social support and increase depressed mood [102]. People living with HIV who had a high level of social support reported less HIV-related stigma [31].

### 4.3. Limitations of the Study

This study aimed to examine the factors of depression in a study area with no prior evidence on the prevalence and associated factors. Even though the study used primary data on the level of depression and related factors, including mediation analysis of social support as a mediator between perceived HIV-related stigma and depression with trained data collectors, pretested questionnaires, and supervisors, the results must be interpreted in light of the following limitations.

First, since the study conducted was cross-sectional, it is difficult to infer a causal relationship. Second, study participants were assessed using a self-reported tool for assessing depressive symptoms rather than the gold standard of screening clinical interviews for major depressive disorder. Third, since this was a health facility-based study, the findings may not be generalizable to the general population. Fourth, data regarding perceived HIV-related stigma and social support use were collected through self-report questionnaires, which might be affected by social desirability bias (the study participants tend to give responses that they think will be perceived positively by others, rather than reflecting their genuine thoughts or behaviors). The other bias was recall bias, in which study participants do not accurately remember past experiences or events. The constraints of using a self-report measure for depression significantly affect the accuracy and reliability of depression assessment in both research and clinical settings. The final limitation is that mediation analysis using the Baron and Kenny method is susceptible to bias, which can introduce potential confounding factors that distort the estimates of mediation effects.

## 5. Conclusion

The magnitude of depression among WLWHIV in health facilities in Gondar town was higher. More than two out of every five WLWHIV had depression. Being single, divorced, widowed, having comorbid medical illness, having a high viral load, and experiencing psychological violence were significant factors for depression. Perceived HIV-related stigma and food insecurity increased depression, whereas increased social support decreased depression in WLWHIV. Social support was a mediator of perceived HIV-related stigma and depression. To address issues related to depression, healthcare providers pay special attention to depression screening and related factors. Then, after giving intervention, if a referral is necessary, they were referred to the clinic where the psychiatric service is provided. Counseling programs must be strengthened, especially for women who are single, widowed, or experiencing psychological violence. Strengthening social support and minimizing perceived HIV-related stigma and counseling in food insecurity would also be needed to support WLWHIV and depression.

### 5.1. Implications

These findings are pivotal because they inform the need to develop public health initiatives aimed at improving depression in WLWHIV. This study reminds clinicians, psychiatric professionals and social service providers that stigma remains a threat to improving depression in WLWHIV. As this was a cross-sectional study, we recommend that future researchers consider undertaking longitudinal and interventional studies to investigate how to effectively mitigate depression and related factors in Ethiopia and in similar settings.

## Figures and Tables

**Table 1:** Sociodemographic characteristics of WLWHIV in Gondar town, Northwest Ethiopia, 2023 (*N* = 1043).

Variable		Frequency	Percentage
Age	Mean with standard deviation 41.41(10.49)		

Marital status	Single	101⁣^∗^	9.7
	Married	330	31.6
	Divorced	**385**	**36.9**
	Widowed	227	21.8

Religious	Orthodox	**929**	**89.1**
	Protestant	16	1.5
	Muslim	86	8.2
	Catholic	9	0.9
	Seven-day Adventist	3⁣^∗^	0.3

Income category	Low income	**689**	**66.1**
	Middle income	214	20.5
	High income	140⁣^∗^	13.4

Educational level	No formal education	**394**	**37.8**
	Primary school	275	26.4
	Secondary school	267	25.6
	Diploma and above	107⁣^∗^	10.3

Residency	Urban	**980**	**94.0**
	Rural	63	6.0

Living arrangement	Alone	139	13.3
	Family	**896**	**85.9**
	Relatives	8⁣^∗^	0.8

Distance of the institution	5 km and less	418	40.1
	More than 5 km	**625**	**59.9**

*Note:* Numbers displayed in bold font indicate high frequency. Numbers with ⁣^∗^ indicate low frequency.

**Table 2:** Obstetric factors among women living with HIV in Gondar town, Northwest Ethiopia, 2023 (*N* = 1043).

Variable		Frequency	Percentage
Unplanned pregnancy	Yes	51	4.9
	No	992	95.1

History of infertility	Yes	37	3.5
	No	1006	96.5

History of still birth	Yes	13	1.2
	No	1030	98.8

History of abortion	Yes	74	7.1
	No	969	92.9

Premature baby	Yes	9	0.9
	No	1034	99.1

Number of children	Do not have a child	179	17.2
	1 to 2 children	503	48.2
	3 to 4 children	265	25.4
	5 and above children	96	9.2

**Table 3:** Clinical factors of the respondents among women living with HIV in Gondar town, Northwest Ethiopia, 2023 (*N* = 1043).

Variables		Frequency	Percentage
ART side effect	Yes	17	1.6
	No	1026	98.4

COVID-19 fear	Yes	317	30.4
	No	726	69.6

HIV disclosure	Yes	750	71.9
	No	293	28.1

ART starting duration	Less than 5 years	142	13.6
	More than 5 years	901	86.4

**Table 4:** Bivariate and multivariate logistic regression analysis of several variables and depression among women living with HIV in Gondar town, Northwest Ethiopia, 2023 (*N* = 1043).

Variables		Depression		COR with 95% CI	AOR
		Yes	No		
Marital status	Married	108	222	1	1
	Single	40	61	1.35 (0.85–2.14)	**1.80 (1.09–2.99) **⁣^∗^
	Divorced	175	210	1.71 (1.26–2.32)	**1.56 (1.11–2.19) **⁣^∗∗^
	Widowed	112	115	2.00 (1.42–2.83)	**1.93 (1.31–2.84) **⁣^∗∗∗^

Unplanned pregnancy	Yes	28	23	1.75 (0.99–3.08)	1.48 (0.77–2.84)
	No	407	585	1	1

History of infertility	Yes	21	16	1.88 (0.97–3.64)	1.25 (0.60–2.62)
	No	414	592	1	1

History of abortion	Yes	39	35	1.61 (1.00–2.59)	1.16 (0.67–2.00)
	No	396	573	1	1

Medical illness	Yes	68	40	2.63 (1.74–3.97)	**2.74 (1.75–4.30) **⁣^∗∗∗^
	No	367	568	1	1

Family history of mental illness	Yes	19	11	2.48 (1.17–5.26)	1.50 (0.65–3.43)
	No	416	597	1	1

Viral load	Less than 75 copies/mL	405	584	1	1
	75 copies/mL and more	30	24	1.80 (1.04–3.13)	**1.86 (1.00–3.45) **⁣^∗^

Fear of coronavirus	Yes	160	157	1.67 (1.28–2.18)	1.32 (0.98–1.79)
	No	275	451	1	1

Disclosed HIV status	Yes	328	422	1.35 (1.02–1.78)	1.32 (0.96–1.81)
	No	107	186	1	1

Duration of ART medication	Mean 11.28 (SD = 4.97)			1.026 (1.00–1.05)	1.01 (0.98–1.04)

Alcohol use for the last 3 months	Yes	174	172	1.69 (1.30–2.19)	1.23 (0.89–1.65)
	No	261	436	1	1

ART adherence	Yes	337	508	1	1
	No	98	100	1.48 (1.08–2.02)	0.93 (0.65–1.32)

Social support	Mean 9.17 (SD ± 2.24)			0.84 (0.79–0.89)	**0.90 (0.84–0.96) **⁣^∗∗∗^

HIV-related stigma	Mean 24.38 (SD ± 6.53)			1.06 (1.04–1.08)	**1.04 (1.02–1.06) **⁣^∗∗∗^

Food insecurity	Mean 3.25 (SD ± 4.22)			1.12 (1.08–1.15)	**1.07 (1.03–1.11) **⁣^∗∗∗^

Physical violence	Yes	98	52	3.11 (2.16–4.47)	1.55 (0.93–2.58)
	No	337	556	1	1

Psychological violence	Yes	144	80	3.27 (2.40–4.45)	**2.05 (1.29-3.23) **⁣^∗∗^
	No	291	528	1	1

Sexual violence	Yes	30	22	1.97 (1.12–3.47)	0.69 (0.35–1.33)
	No	405	586	1	1

Abbreviations: AOR, adjusted odds ratio; CI, confidence interval; COR, crude odds ratio.

⁣^∗^*p* value < 0.05.

⁣^∗∗^*p* value < 0.01.

⁣^∗∗∗^*p* value < 0.001.

**Table 5:** Mediation analysis among social support, stigma, and depression among women living with HIV in Gondar town, Northwest Ethiopia, 2023.

Effect	Path	*β*	SE	95% CI		*p* value
				Lower	Upper	
Total	HIV-related stigma-> depression	0.17	0.0386	0.0971	0.2484	< 0.001
Indirect effect	HIV-related stigma-> social support-> depression	0.04	0.0095	0.0235	0.0607	< 0.001
Direct effect	HIV-related stigma-> depression	0.13	0.0291	0.0736	0.1877	< 0.001

## Data Availability

The dataset during and/or analyzed during the current study is available from the corresponding author on reasonable request.
